# Risk factors for postoperative retears in patients with small to medium tears following arthroscopic rotator cuff repair

**DOI:** 10.1016/j.jseint.2025.101610

**Published:** 2025-12-26

**Authors:** Kazuki Nishida, Hiroki Ohzono, Masafumi Gotoh, Ryunosuke Abe, Hiroaki Moriyama, Hidehiro Nakamura, Yasuhiro Mitsui, Koji Hiraoka, Takahiro Okawa

**Affiliations:** a Department of Orthopaedic Surgery, Kurume University Medical Center, Fukuoka, Japan; b Gotoh Orthopaedic Clinic, Kumamoto, Japan; c Department of Orthopaedic Surgery, Hyakutake Orthopaedic Hospital, Saga, Japan; d Department of Orthopaedic Surgery, Kurume University Hospital, Fukuoka, Japan

**Keywords:** Small/medium cuff tears, Risk factor, Arthroscopic rotator cuff repair, Retear rate

## Abstract

**Background:**

The retear rate after arthroscopic rotator cuff repair for small/medium tears is considered lower than that for large/massive tears. However, few studies have reported risk factors for small/medium retears. Therefore, this study investigated the risk factors for small/medium retears following arthroscopic rotator cuff repair.

**Methods:**

The retrospective study involved 129 patients who underwent arthroscopic rotator cuff repair for small/medium rotator cuff tears at our hospital between January 2018 and June 2021. Clinical evaluations at preoperative and final follow-up were conducted using the criteria of University of California, Los Angeles score and Constant Shoulder score, and the achievement of minimal clinically important difference of those scores were recorded. Physical examination findings, imaging findings (stump classification and Goutallier classification), suturing methods, and past medical history including smoking history and diabetes were investigated to assess their relevance to clinical outcomes and retear. At the final postoperative follow-up, structural outcome was evaluated using magnetic resonance images, and Sugaya classification type 4 or 5 was defined as a retear.

**Results:**

In total, 76 male and 53 female patients with a mean age of 64.2 ± 9.1 years were enrolled. The shortest follow-up period was 12 months postoperatively (range, 12–44 months; mean, 22.09 ± 7.27 months). All clinical scores of 129 patients showed significant postoperative improvement (*P* < .001). Based on the Cofield classification, 44 patients had small tears compared with 85 patients who had medium tears, whereas 15 (11.6%) patients experienced retears. Multivariate analysis of retear-related factors revealed that symptom duration, absence of traumatic episode, tendon edge status (stump classification), Goutallier classification of the supraspinatus (stages ≥2), and absence of the long head of the biceps tendon during surgery were significant risk factors for retears (*P* < .01). The correlation analysis between symptom duration and other factors showed a significant association with traumatic absence only (*P* < .0001).

**Conclusion:**

This study showed that after arthroscopic rotator cuff repair for small/medium rotator cuff tears, patients are exposed to a substantial number of risk factors of postoperative retears of which symptom duration and absence of traumatic episodes were the only inter-related factors.

Rotator cuff tears frequently occur in middle-aged and older individuals, which cause severe shoulder pain with restricted range of motion (ROM).^4^^,^^9^ The main causes of the tears are degenerative changes of the tendon, and their frequency drastically increases among individuals aged >50 years.^9^ The conservative approaches are commonly employed as the first choice of treatment, which include intra-articular injection, medication, and rehabilitation.^17^ The failure of conservative treatment often leads to surgical intervention such as arthroscopic rotator cuff repair (ARCR), as an alternative option. Although the clinical outcomes after ARCR are acceptable, postoperative retears are a significant issue postoperatively.^24^

The age of individuals, tear size, muscle fatty degeneration, and tendon edge degeneration have been reported as risk factors affecting postoperative retears.^7^^,^^8^^,^^11^^,^^24^ According to the Goutallier classification, intramuscular fatty degeneration is categorized into 5 stages by magnetic resonance imaging (MRI), and the retear rate increases with the increasing percentage of the fatty degeneration in the muscles.^12^^,^^13^ Ishitani et al advocated stump classification for evaluating the association between degeneration of tendon edges and postoperative retears.^5^ They defined the tendon edge status into 3 types by measuring the signal intensity of the tendon edge relative to the deltoid (types 1–3) and demonstrated that a type 3 tendon edge was the most significant sign for postoperative retears.^8^

Several studies have reported higher retear rates in large to massive cuff tears. A meta-analysis study demonstrated a retear rate of 37% in large/massive tears and concluded that larger tears were associated with higher retear rates.^10^ Yoo et al showed that the overall retear rate of arthroscopically repaired large/massive rotator cuff tears was 45.5%. However, they specifically dealt with large/massive tears that were repaired completely in which <50% of the medial-to-lateral footprint was covered. Bishop et al reported a retear rate of 76% with 1-year MRI follow-up in patients with large/massive rotator cuff tears following arthroscopic repair.^1^

Regarding small/medium cuff tears, the retear rates are reported to be 5%–10.3% in the previous literature.^14^^,^^20^ Although the retear rates in small/medium cuff tears are relatively low, few studies have evaluated the risk factors for postoperative retears. Therefore, this study sought to identify the risk factors for postoperative retears following ARCR in patients with small/medium cuff tears.

## Materials and methods

### Patients

**Figure 1:**
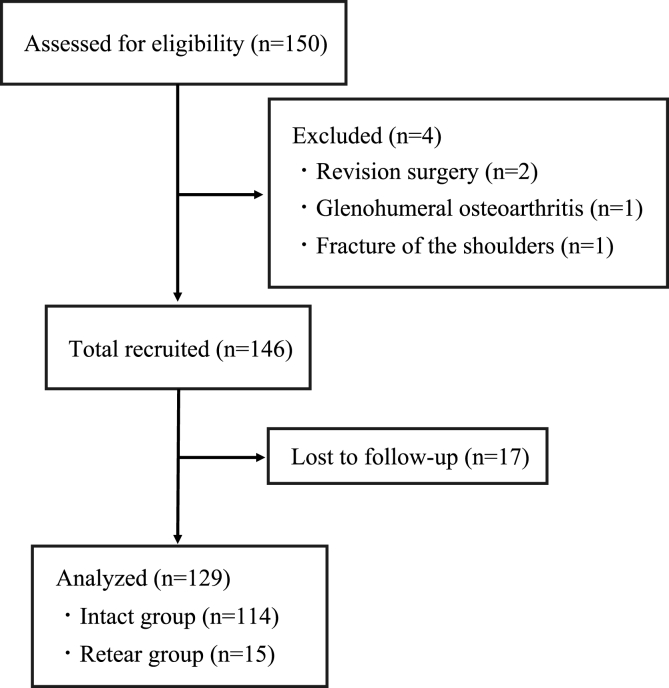
Flowchart of this study according to the Consolidated Standards of Reporting Trials guidelines.

Participants for this retrospective study were 150 consecutive patients who presented with small to middle cuff tears and underwent ARCR in our institute. Patients who had complete footprint coverage during surgery and those who were followed up for at least 1 year, with an average follow-up period of 22 ± 7.3 months, were included. Conversely, patients with advanced glenohumeral arthritis, fractures of the shoulders, revision surgeries, or lost to follow-up were excluded. Consequently, 129 patients met our criteria, excluding 19 patients who were lost to follow-up (Fig. 1). The patients consisted of 78 males and 53 females, with a mean age of 64.2 ± 9.1 years. Based on DeOrio and Cofield classification, the patients had 86 small and 45 medium cuff tears. Details are shown in detail in Table I.

### Surgical procedure and postoperative regimen

All surgeries were performed under general anesthesia with the patient in the beach-chair position by 1 of 4 trained shoulder surgeons. The torn cuff was repaired using the single-row or suture bridge technique depending on tendon mobility and tear configuration. For single-row repairs, 1 row of anchors (HEALCOIL, Smith & Nephew, El Coyol, Costa Rica) was placed on the lateral aspect of the footprint, and the torn cuff was fixed with interrupted sutures. For suture bridge repair, 1 row of anchors was placed on the medial aspect of the footprint and the torn cuff was transosseously fixed with the knotless anchor on the lateral aspect of the footprint (SwiveLock, Arthrex, Naples, FL, USA). Additional procedures including capsular release, tenotomy/tenodesis of the long head of the biceps (LHB) tendon, and acromioplasty, were performed as needed.

Postoperatively, the patients were immobilized in a sling with an abduction pillow, with the shoulder internally rotated at 30°–40° and abducted at 20°. Passive ROM exercises of the shoulder commenced on day 1 postoperatively, and active ROM exercises were allowed at week 1 postoperatively. Isometric muscle strengthening exercises were allowed at week 4 postoperatively, and isotonic exercises were started at week 9 postoperatively.

### Functional outcomes

University of California, Los Angeles (UCLA), and Constant Shoulder (CS) scores were used as clinical outcome measures, and the achievement of the minimal clinically important difference (MCID) for each score was recorded. ROM was assessed with a goniometer, and visual analog scale scores at 3 states (at rest, at shoulder motion, and at night) were reported as patients' subjective assessments of pain. These measures were evaluated preoperatively and postoperatively by a physical therapist blinded to this study.

### Structural outcome

Tear size, fatty degeneration, preoperative muscle atrophy, and postoperative structural integrity were examined using MRI according to the following criteria: postoperative “intact tendons” were defined as types I–III per the Sugaya classification.^20^ The fatty degeneration of the rotator cuff muscles at the Y-view was evaluated according to the Goutallier classification.^15^ According to the methods developed by Ishitani et al,^5^ the signal intensities in the oblique–coronal view of T2-weighted fat-suppressed images were measured at 5–8 mm from the tip of the tendon stump (C) and in the deltoid (D). The relative ratio of C to D was measured and classified to types 1–3: C/D < 0.8 as type 1, 0.8–1.3 as type 2, and >1.3 as type 3.

### Statistical analysis

JMP15 (SAS Institute, Cary, NC, USA) was used for statistical analysis. The Wilcoxon test was used to compare the preoperative and postoperative UCLA scores. In the univariate analysis, unadjusted *P* values were calculated using logistic regression analysis to examine the relationships between the clinical parameters in intact and the retear groups, and Benjamini–Hochberg (BH) adjusted *P* values were then calculated to control the false discovery rate for multiple comparisons. For the subsequent multivariate logistic regression analysis to identify the independent factors associated with retear, variables with an unadjusted *P* value ≦ .1 in univariate analysis instead of BH-adjusted *P* value were included in order to retain a broader range of factors in this exploratory analysis. Odds ratio (OR) with a 95% confidence interval (CI) were also calculated. Receiver operating characteristic (ROC) curve analysis was performed to obtain the cutoff value of symptom duration, ROM of elevation, ROM of abduction, Goutallier stages of the supraspinatus, and stump classification affecting postoperative retears. The cutoff value was determined as the maximum value of the Youden index [sensitivity 1 (1 2 specificity)] on the ROC curve. The data were expressed as the mean values with standard deviation. A *P* value <.05 was considered significant.

## Results

Overall, 129 patients were included in this study. The mean age of the patients at surgery was 64.2 ± 9.1 years, and the mean follow-up period was 22 ± 7.3 months. Forty-eight patients (37.2%) had traumatic tears had traumatic tears with a clear injury history, whereas 81 patients (62.8%) had degenerative tears without trauma. The mean symptom duration before surgery was 10.7 ± 22.5 months.

### Functional outcome

**Figure 2:**
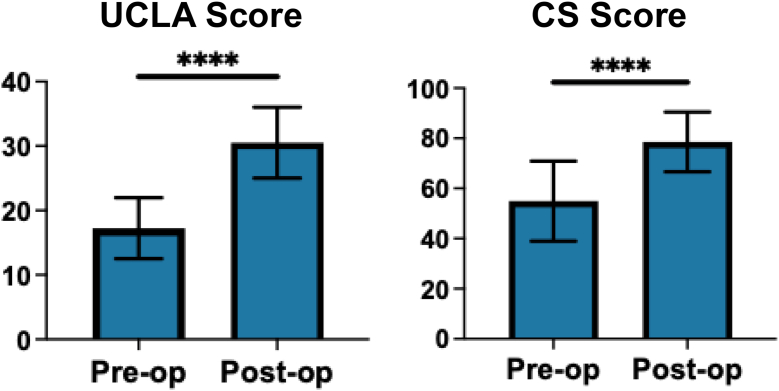
Clinical outcome of UCLA score and CS score. ∗∗∗∗*P* < .0001. *UCLA*, University of California, Los Angeles; *CS*, Constant Shoulder.

The functional score of all patients significantly improved from 17.2 ± 4.7 points preoperatively to 30.5 ± 5.5 points postoperatively in the UCLA score (*P* < .0001) and 54.7 ± 16.1 points preoperatively to 78.4 ± 11.9 points postoperatively in the CS score (*P* < .0001) (Fig. 2). No significant difference was found in the mean preoperative UCLA scores and CS scores between patients with satisfactory and unsatisfactory outcomes (*P* = .39) (Table I). The achievement rates of MCID were 86.0% for UCLA score and 79.8% for CS score at final follow-up. In UCLA score, 87.7% of patients without retear achieved MCID compared with 73.3% of those with retear. (*P* = .17) and in CS score, 81.6% of patients without retear achieved MCID compared with 66.7% of those with retear in CS score (*P* = .16) (Table II).

**Table I:** Comparison of clinical variables between the intact and retear groups.

Variables	Total	Intact	Retear	*P* value	Benjamini–Hochberg adjusted *P* value
Preoperative demographic variables					
Age, yr	64.2 ± 9.1	64.4 ± 9.4	62.6 ± 6.9	.23	.79
Sex, male/female, n	76/53	67/47	9/6	.9	.96
Symptom duration, mo	9 ± 10	7.5 ± 7.2	20.3 ± 18.3	.002	<.001
Diabetes, n (%)	22 (17)	19 (17)	3 (20)	.75	.90
Hypertension, n (%)	48 (37)	43 (38)	5 (33)	.74	.90
Smokers, n (%)	39 (30)	34 (30)	5 (33)	.78	.90
Traumatic onset, n (%)	48 (37)	47 (41)	1 (7)	.009	.051
Workers' compensation, n (%)	9 (7)	8 (7)	1 (7)	.96	.96
Traffic accident, n (%)	11 (9)	18 (16)	3 (19)	.75	.32
Follow-up, mo	22.1 ± 7.3	22.3 ± 7.4	20.5 ± 6.6	.41	
Preoperative functional variables					
Range of motion, degrees					
Elevation	119 ± 33.7	116 ± 34.6	140 ± 13.6	.046	.051
Abduction	108 ± 45.2	104 ± 45.8	137 ± 27.8	.008	.051
Internal rotation	67 ± 7.5	67 ± 7.2	64 ± 9.5	.27	.34
External rotation	44 ± 19.1	44 ± 18.6	47 ± 23.5	.27	.80
UCLA score	17.3 ± 4.7	17.2 ± 4.9	17.9 ± 3.2	.62	.80
Constant score	54.9 ± 16	54 ± 16.6	62.4 ± 6	.07	.27
Visual analog scale, mm					
Rest	12.6 ± 19.7	12.1 ± 18.9	16 ± 25.3	.28	.79
Motion	52.7 ± 29.2	51.6 ± 29.1	61.6 ± 29.2	.25	.50
Night	41.1 ± 33.4	40.1 ± 33.5	48.2 ± 32.8	.44	.76
Preoperative structural variables					
Tear size, n (%)				.52	.79
Small	44 (34)	40 (35)	4 (27)		
Medium	85 (66)	74 (65)	11 (73)		
Goutallier classification, n (%)					
Supraspinatus, n (%)				.11	.34
Stage 0	35 (27)	30 (26)	5 (33.3)		
Stage 1	58 (45)	55 (48)	3 (20)		
Stage 2	30 (23)	25 (22)	5 (33.3)		
Stage 3	6 (5)	4 (4)	2 (13.3)		
Stage 4	0	0	0		
Infraspinatus, n (%)				.92	.90
Stage 0	97 (75)	85 (74.6)	12 (80)		
Stage 1	29 (22)	26 (22.8)	3 (20)		
Stage 2	2 (2)	2 (1.8)	0		
Stage 3	1 (1)	1 (0.9)	0		
Stage 4	0	0	0		
Subscapularis, n (%)				.83	.90
Stage 0	105 (81)	92 (81)	13 (87)		
Stage 1	23 (18)	21 (18)	2 (13)		
Stage 2	1 (1)	1 (1)	0		
Stage 3	0	0	0		
Stage 4	0	0	0		
GFDI	0.51	0.51 ± 0.47	0.53 ± 0.52	.9	.90
Stump Classification, n (%)				.0087	.051
Type 1	18 (14)	18 (16)	0 (0)		
Type 2	43 (33)	41 (36)	2 (13)		
Type 3	67 (52)	55 (48)	12 (80)		
Intraoperative Variables					
Intraoperative long head biceps tendon, n (%)				.09	.34
Absent	11 (9)	8 (7)	3 (20)		
Present	118 (91)	106 (93)	12 (80)		
Treatment of LHB, n (%)				.02	.27
Preserve	56 (43)	48 (42)	8 (53.3)		
Tenotomy	69 (54)	64 (56)	5 (33.3)		
Tenodesis	4 (3)	2 (2)	2 (13.3)		
Repair technique, n (%)				.33	.67
Single-row	29 (23)	24 (21)	5 (33)		
Suture bridge	99 (77)	89 (79)	10 (67)		

*LHB*, long head of the biceps; *UCLA*, University of California, Los Angeles; *GFDI*, Global Fatty Degeneration Index.

**Table II:** Achievement of the MCID at the final follow-up.

Risk factors	Threshold	Total n (%)	Intact group n (%)	Retear group n (%)	*P* value
UCLA Score	+6	111/129 (86.0%)	100/114 (87.7%)	11/15 (73.3%)	.17
Constant Shoulder Score	+10.4	103/129 (79.8%)	93/114 (81.6%)	10/15 (66.7%)	.16

*UCLA*, University of California, Los Angeles; *MCID*, minimal clinically important difference.

### Postoperative retears

According to the Sugaya classification, 15 (11.6%) patients were assigned to retear group and 114 (88.4%) patients were assigned to intact group. The Goutallier and stump classifications of these patients are shown in Table I.

### Univariate analysis to detect the factors affecting postoperative retears

The results of the univariate analysis showed that preoperative involved periods (*P* = .002), the absence of traumatic history (*P* = .009), ROM of elevation (*P* = .0046), ROM of abduction (*P* = .008), and Stump classification (*P* = .0087) were significantly associated with postoperative retears, and only preoperative involved periods remained significant after the BH adjustment procedure. Details are shown in Table I.

### Multivariate analysis to detect factors affecting postoperative retears

Before the multivariate analysis, the cutoff values of preoperative involved periods, ROM of elevation, ROM of Abduction, Goutallier stage of the supraspinatus, and stump types were evaluated using the ROC curve. Consequently, 7 months before the surgery, 125° of elevation, 100° of abduction, Goutallier stage 2, and type 3 stumps were obtained as the cutoff values for postoperative retear.

The multivariate logistic regression analysis using variables with unadjusted *P* value ≦.1 in univariate analysis showed that symptom duration >7 months (OR 9.51, 95% CI 1.24–72.8, *P* = .03), absence of traumatic history (OR 9.82, 95% CI 1.87–180.9, *P* = .04), absence of LHB during surgery (OR 95.1, 95% CI 2.8–3231.7, *P* = .01), and type 3 stump (OR 34.9, 95% CI 3.31–367.3, *P* = .003) were significantly associated with postoperative retear. Details are shown in Table Ⅲ.

**Table III:** Results of multivariate logistic regression analysis.

Risk factors	OR	LL 95% CI	UL 95% CI	*P* value
Symptom duration >7 mo	9.51	1.24	72.8	.03
Absence of traumatic history	9.82	1.87	180.9	.04
Preoperative ROM of elevation <125	1.59	0.2	12.4	.66
Preoperative ROM of abduction <100	5.31	0.59	47.8	.14
Absence of LHB	95.1	2.8	3231.7	.01
Goutallier stage of supraspinatus ≧2	3.51	0.67	18.3	.14
Stump type ≧3	34.9	3.31	367.3	.003

*LHB*, long head of the biceps; *CI*, confidence interval; *OR*, odds ratio; *ROM*, range of motion; *UL*, upper limit; *LL*, lower limit.

### Association of the preoperative involved periods with the other clinical variables

The involved periods demonstrated a significant correlation with the absence of traumatic episode (*P* = .0011) but not with Goutallier stages, LHB absence during surgery, and stump classification.

## Discussion

This study examined the risk factors for postoperative retears in patients with small/medium cuff tears following ARCR. Postoperative retears were seen in 11.6% (15 of 129 patients) in our series. Under these conditions, the multivariate logistic regression analysis showed that the involved periods (>7 months), absence of traumatic history, absence of LHB during surgery and type 3 stump were significantly associated with postoperative retears.

Few studies have evaluated the factors for postoperative retear after ARCR especially in small/medium tears. Park et al suggested that higher than stage Ⅱ infraspinatus fatty degeneration, larger than 2 cm tear size and older than 69 years correlated with the retear after ARCR in small/medium tear.^16^ Kawamata et al suggested that tear size in medial-lateral width and C/D ratio were risk factors for the retear after ARCR in medium tear.^6^ Inconsistent with those previous studies, tear size, older age, and infraspinatus fatty degeneration were not significantly associated with the retear in the present study.

Boughebri et al assessed 46 patients with small supraspinatus tears treated arthroscopically.^2^ In their study, 13 patients had postoperative retears (28.2%), with an average of 35 months postoperatively, proposing comparable retear rates in arthroscopic cuff repair series of the single-tendon supraspinatus tears (20.4%–39%). Unlike these results, several studies have reported better structural outcomes. Park et al demonstrated that in 339 patients with arthroscopic cuff repair, 45 had retears at 1 year postoperatively (13.3%).^16^ Neyton et al showed that in small/medium supraspinatus tears treated by suture bridge repair, 10.3% had postoperative retears.^14^ Similarly, postoperative retears were noted in the present series (11.6%).

Kim In-Bo and Kim Moo-Won revealed that in full-thickness cuff tears treated by arthroscopic suture bridge technique, longer duration of involvement was significantly associated with postoperative retears.^8^^,^^10^ A recent meta-analysis also demonstrated that patients whose symptoms lasted longer had significantly higher retear rates 6 months after ARCR.^24^ Consistent with the findings of the aforementioned studies, the present study showed that in small/medium cuff tears, the involved periods are significantly associated with postoperative retears.

Ishitani et al demonstrated a significant association of the tendon edge status at the repair site with tendon healing.^5^ They defined the 3 types of tendon edge status by measuring the signal intensity of the tendon edge relative to the deltoid (types 1–3). Consequently, type 3 stumps had significantly higher retear rates, suggesting that the stump's signal intensity may be an important indicator for assessing its condition. Shinohara et al further confirmed stump type 3 is associated with retear following ARCR with suture bridge and double-row repair, and Takeuchi et al showed that the machine learning model including stump classification as one of the parameters accurately predicted retear after ARCR.^19^^,^^21^ A basic study revealed that type 3 stumps had a higher accumulation of advanced glycation end products, increased oxidative stress and associated apoptosis, and reduced cell viability, resulting in rotator cuff fragility.^18^^,^^23^ The present study consistently showed that type 3 stumps significantly affect postoperative tendon retears in small/medium cuff tears.

Tan et al demonstrated that the preoperative symptom duration affected retear rates in the traumatic group (*P* = .014) but not in the nontraumatic group.^22^ They concluded that the preoperative symptom duration may be important in surgical repairs of traumatic rotator cuff tears. In the present study, the postoperative retear rates were significantly higher in patients with a nontraumatic episode than those with a traumatic episode; in this series, preoperative symptom duration exhibited a significant association with the absence of traumatic episodes. Thus, these results indicate that symptom duration is relatively associated with postoperative retears rather than traumatic episodes.

In their systematic review, Zhao et al reported that intraoperative biceps lesion/procedure directly affected rotator cuff retears following ARCR.^24^ They also emphasized the need for further study to determine the mechanism of this action given the scarcity of studies on this aspect. In the present study, the intraoperative absence of the biceps tendon was significantly associated with postoperative retears in small/medium cuff tears but not with preoperative symptom duration. This remains to be clarified in the future study.

The present study has several limitations. First, its retrospective design may have introduces potential selection bias and limits the accuracy and completeness of clinical data. Second, variables for multivariate analysis were selected based on unadjusted *P* value rather than BH-adjusted *P* values to explore a broader range of potential factors, which increases the risk of false-positive findings. Therefore, findings of this study should be regarded as hypothesis-generating rather than conclusive. Third, the factors previously reported to affect the retear rates—such as preoperative steroid injection history, bone mineral density, critical shoulder angle, and acromiohumeral distance—were not evaluated in this study.^3^^,^^24^ Forth, multiple surgical procedures were performed based on the individual surgeon's decisions that introduce variability and may influence the outcomes.

## Conclusion

This study focused on patients with small/medium cuff tears following ARCR and provided important information on postoperative retears in these patients. The results revealed that symptom duration (>7 months), absence of traumatic history, absence of LHB during surgery, and type 3 stump were significantly associated with postoperative retears. Orthopedic physicians should be aware of these risk factors when performing ARCR, particularly in patients with small/medium cuff tears.

## Supplementary Data

STROBE Checklist
